# Mapping evidence on the prevalence, incidence, risk factors and cost associated with chronic low back pain among adults in Sub-Saharan Africa: a systematic scoping review protocol

**DOI:** 10.1186/s13643-020-01321-w

**Published:** 2020-03-17

**Authors:** Morris Kahere, Themba Ginindza

**Affiliations:** Discipline of Public Health Medicine, School of Nursing and Public Health, University of KwaZulu-Natal, 2nd Floor George Campbell Building, Mazisi Kunene Road, Durban, 4041 South Africa

**Keywords:** Chronic low back pain, Prevalence, Incidence, Mortality, Cost-of-illness, Risk factors, Disability

## Abstract

**Background:**

Globally, low back pain (LBP) is a major public health problem affecting mainly adults of the working class and is the leading cause of disability. The estimated lifetime prevalence of LBP is 50 to 80%. From 1990 to 2015, the years lived with disability caused by LBP have scaled up by 54% with the greatest increase observed in low-middle-income countries (LMICs). LBP poses a significant socio-economic burden to the society regardless of all the technological advancement in diagnosis and intervention approaches in recent years. Despite an increase in the literature of LBP in LMICs, chronic low back pain (CLBP) is poorly investigated yet it is responsible for the largest amount of burden. The purpose of this scoping review is to map the existing evidence on the prevalence, incidence, mortality, risk factors, and cost associated with CLBP among adults in Sub-Saharan Africa (SSA).

**Methods:**

We will conduct a scoping review to explore, describe, and map literature on the prevalence, incidence, mortality, risk factors, and costs associated with CLBP among adults in SSA. The search will be performed using the EBSCOhost platform by searching the following databases within the platform: Academic search complete, health source: nursing/academic edition, CINAHL with full text, Embase, PubMed, MEDLINE, Science Direct databases, Google Scholar, and the World Health Organization library databases. The search will include peer-reviewed, review articles, and gray literature. The first (title and abstract) and the second (full text) screening phases will be performed by two independent reviewers, with the third reviewer employed to adjudicate discrepancies. The reference list of all included articles will also be searched for eligible articles. This scoping review will be reported in accordance to the MOOSE and PRISMA-ScR guidelines. The NVivo 12 data analysis software will be used to generate themes, and a thematic content analysis will be used to give the narrative account of the review.

**Discussion:**

The study anticipates finding relevant literature on the prevalence, incidence, risk factors, mortality, and cost associated with CLBP among adults in SSA. The study outcomes will aid in identifying research gaps, planning, informing policy, commissioning of future research, and funding prioritization.

## Background

According to Hoy et al., low back pain (LBP) is the most prevalent among a variety of other musculoskeletal disorders with an estimated global lifetime prevalence of 70–80%, a 1-year prevalence of 15–45% and an average point prevalence of 30% among the general population [1]. A systematic review by Morris et al. investigating the prevalence of low back pain in Africa revealed that the lifetime, 1-year and point prevalence of low back pain among African populations was substantially higher than the revealed global LBP prevalence estimates [2]. Morris et al. found that the point prevalence of low back pain among African countries was 39%, while the global point prevalence according to Hoy et al. was 18.3% [1, 2]. Similarly, the 1-year prevalence of low back pain among Africans (57%) was significantly higher than the global annual prevalence estimates (38.5%) [1, 2]. According to Hoy et al. and Majid et al., a higher prevalence of low back pain has been significantly correlated with a low socio-economic status and lower educational levels [1, 3]. Low back pain possesses a significant socio-economic burden to the society and has been reported to be the leading cause of activity limitation worldwide [4]. Years lived with disability caused by low back pain has scaled up by 54% from the years 1990 to 2015 due to aging and population increase, and the greatest burden thereof has been observed in low-middle-income countries [5]. The burden attributed to low back pain is predicted to continue to increase in the coming decades, particularly in LMICS where the healthcare infrastructure and a conglomeration of other systems are poor equipped to cope with the increasing burden of low back pain in addition to other priorities such as infectious diseases [5].

Low back pain is often defined as pain, muscle tension, or stiffness localized below the coastal margin and above the inferior gluteal fold with or without leg pain [6]. Low back pain is classified as acute (symptoms that lasts less than 3 months) or chronic (symptoms that lasts more than 3 months) based on the duration of symptoms and as specific (known causative factors) or non-specific (idiopathic or unknown origin) based on the etiology of symptoms [7]. About 90% of the low back pain cases will improve in 6 weeks or less, and only about 10% of the cases will progress to recurrent or chronic low back pain [8]. The significant amount of cost and disability associated with low back pain is attributed to this small percentage group of chronic low back pain sufferers [9]. There is widespread literature of low back pain across all continents with little knowledge of chronic low back pain particularly in LMICs [2].

Low back pain has been regarded as a trivial condition, and its research has not been given priority especially in African countries where the concern has been placed on epidemic infectious diseases such as HIV/AIDS, TB, and Malaria [2]. However, according to the Global Burden of Disease 2015, low back pain was found to be the leading cause of disability and associated with a significant amount of cost [10]. While the current focus on public health research and funding has been shifted towards epidemic diseases, the neglection of low back pain research possesses a blind spot threat to the healthcare system. According to Gore et al., the economic burden attributed to low back pain incorporating both direct and indirect costs range from $84.1 billion to $624.8 billion in the USA [11]. Indirect cost due to lost work productivity was the main contributor to this economic burden accounting for $7.4 billion to $28 billion [11]. However, low back pain is also associated with a significant amount of direct cost which includes healthcare resource utilization. Low back pain is the second most common reason to visit a physician after the common cold, the third most cause of surgical procedures, and the fifth ranking cause of admission to hospital.

Low back pain has been found to directly impair activities of daily living (ADL) with the frail constitution suffering more from the chronic subcategory of back pain avoid performing these ADL mainly due to fear of re-injury or worsening of the symptoms [5]. This fear avoidance belief has been shown to have a strong correlation with chronic low back pain and will eventually exacerbate the symptoms culminating in persistent disabling back pain [12]. Being unable to or avoiding these ADL can lead to weight gain and development or progression of other chronic health conditions, and this will ultimately lead to earlier death [12, 13]. A telephonic survey in the USA showed that the prevalence of chronic low back pain in the interval between 1992 and 2006 has more than doubled [14]. The population of adults is increasing worldwide. Approximately 8% of the world population are adults aged 65 years and above, and it is estimated to continue rising to almost 17% by 2050 [13, 15], thus increasing the socio-economic burden of chronic low back pain since this condition is more prevalent to this age group.

A scoping review of the literature regarding the epidemiology, incidence, mortality, risk factors, and economic burden of chronic low back pain among adults in SSA will be conducted. This scoping review will seek to find the evidence-based research gaps and help inform future research policy. The implementation of interventions directed at preventing and controlling the development of chronic low back pain in SSA and a change of the research focus and funding directions will be encouraged. This scoping review protocol therefore aims to highlight the existing knowledge gap in the distribution of chronic low back pain in SSA among adults with estimates on prevalence, incidence, mortality, risk factors, comorbidities, and the associated socio-economic burden.

## Methodology

### Scoping review

**Fig. 1:**
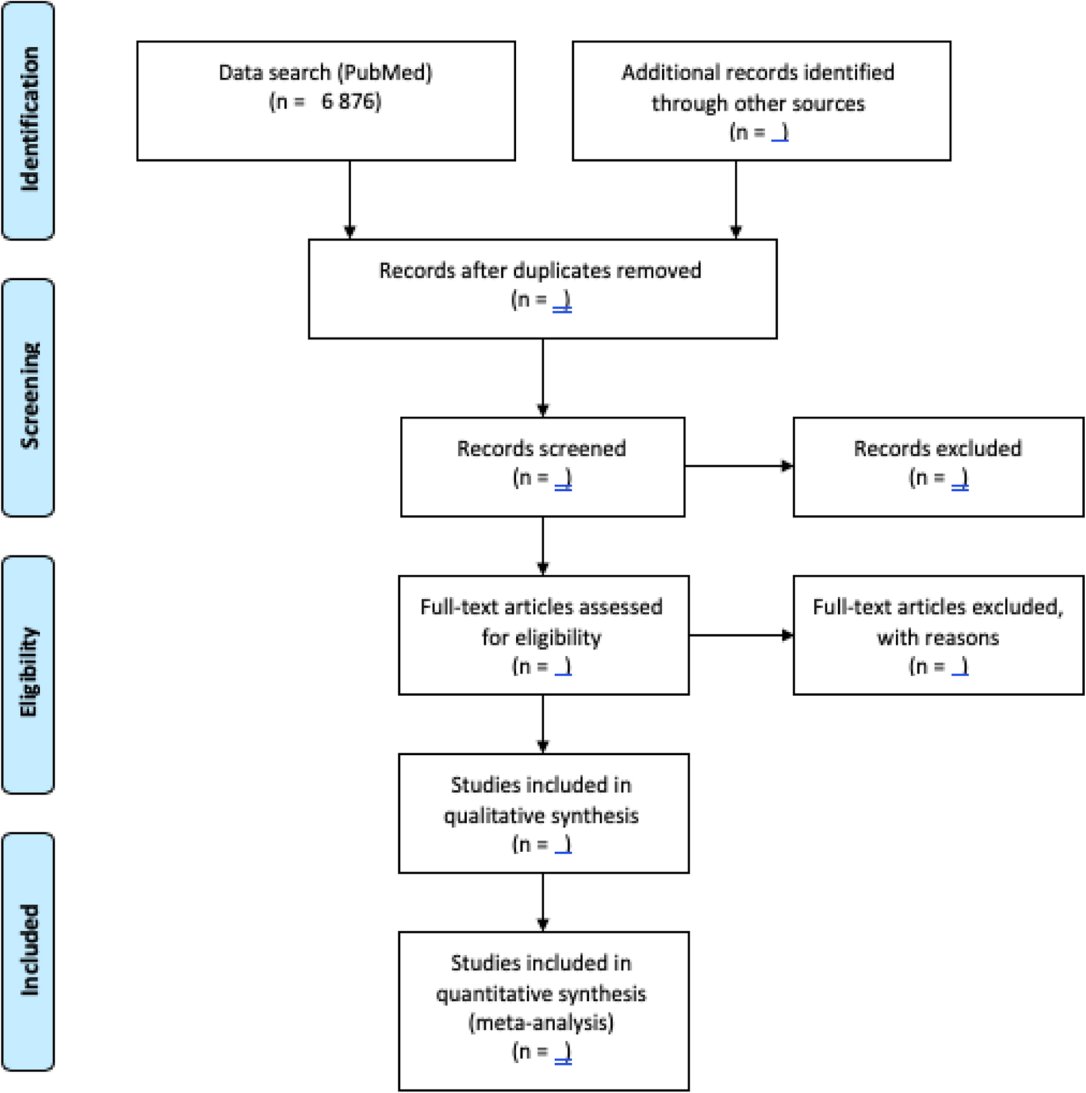
PRISMA-P flow diagram

The proposed scoping review will highlight the available literature or the evidence on the distribution of chronic low back pain among adults in SSA with estimates on prevalence, incidence, mortality, risk factors, comorbidities, and associated cost (economic burden). This protocol is a portion of a large research study, which aims to determine the burden of chronic low back pain in KwaZulu-Natal (a coastal South African Province). A PRISMA flow diagram (Fig. 1) will be utilized to guide the flow of citations reviewed and to illustrate the outcome of the title search from different databases. The proposed scoping review will be reported in accordance with the MOOSE guidelines for observational studies in epidemiology and the preferred reporting items for systematic reviews and meta-analysis extended for scoping reviews (PRISMA-ScR) [16] (Additional file 1). A methodological framework proposed by Arksey and O’Malley will be used [17]. This Arksey and O’Malley methodological framework is identified by the following six steps, (I) Identify the research question, (II) Identify the relevant studies, (III) Study selection, (IV) Charting the data, (V) Collating, summarizing and reporting data, and (VI) Consultation (optional) [17].

#### Identifying the research question

The main research question of the proposed scoping review is, “What is the existing evidence on the distribution of chronic low back pain among adults in SSA?”

1. Sub-questions
  - What is the burden of chronic low back pain in SSA with estimations on the prevalence, incidence and mortality?
  - What are the comorbidities and risk factors associated with chronic low back pain in SSA?
  - What are the estimated costs associated with chronic low back pain?
2. Inclusion criteria (studies that will be included in this review should present evidence on either of the factors listed below)

- The prevalence of chronic low back pain in SSA
- The incidence of chronic low back pain in SSA
- The risk factors associated chronic low back pain in SSA
- The comorbidities associated with chronic low back pain
- Studies done on the adult population aged 18 years and above
- Studies that have a clear definition of low back pain
- Only studies conducted in English and in other languages with an English version will be included into the study

3. Exclusion criteria

- Studies which do not satisfy the above listed criterion will be excluded.
- Studies done on children or adolescence
- Studies done outside the context of SSA
- Clinical trials and intervention-based studies will be excluded
- Studies contacted in other languages other than English and do not have an English version will also be excluded
- Studies that lack a clear definition of low back pain in terms of its anatomical location

4. Eligibility of research question (the eligibility of the research question was determined using the Population Exposure Context Outcome design (PECOd) framework; some part of it has been recommended by the Joanna Briggs Institute 2015 [18]. This framework is outlined in Table 1)

**Table 1:** PECOd framework for eligibility of research question

Criteria	Determinants
Population	Individual with chronic low back pain
Exposure	Chronic low back pain
Context	Sub-Saharan Africa
Outcome	1. Prevalence 2. Incidence 3. Mortality 4. Risk factors 5. Associated costs 6. Comorbidities and disabilities associated
Design	Cohort, cross-sectional studies

#### Identifying relevant studies (literature search)

A keyword search of studies conducted in English will be performed without a date limit to identify the relevant studies. An electronic literature search will be performed using the EBSCOhost platform by searching the following databases within the platform: Academic search complete, health source: nursing/academic edition, CINAHL with full text, Embase, PubMed, MEDLINE, Science Direct databases, Google Scholar, and World Health Organization (WHO) library databases and gray literature to retrieve articles that are relevant to the objective of proposed scoping review, guided by the study inclusion and exclusion criteria. The following keyword terms will be used: low back pain, lumbar pain, spinal pain, musculoskeletal pain, epidemiology, prevalence, incidence, mortality, risk factors, burden, impact, disability, comorbidities, Africa, Sub-Saharan Africa, and Low-Middle-Income-Countries (Table 2). The Boolean apparatus OR and AND will be used to separate the keywords during the literature search. We have contacted a pilot search using the above keyword to determine the feasibility of the search criteria (Table 3). The primary database search will be performed by the principle investigator from the above-mentioned online databases. All the database search outcomes will then be transferred to the Endnote X8- reference management software, which will be used to create a library for this review. Deduplication will follow immediately after the transfer of all the retrieved articles to the Endnote X8 and prior to the initial (title and abstract) phase of screening.

**Table 2:** Pilot search result from PubMed database

Date of search	Search engine used	Keyword search used	Number of articles retrieved
05/03/2020	PubMed	(((((((“low back pain”[MeSH Terms] OR (“low”[All Fields] AND “back”[All Fields] AND “pain”[All Fields]) OR “low back pain”[All Fields]) OR ((“lumbosacral region”[MeSH Terms] OR (“lumbosacral”[All Fields] AND “region”[All Fields]) OR “lumbosacral region”[All Fields] OR “lumbar”[All Fields]) AND spinal[All Fields] AND (“pain”[MeSH Terms] OR “pain”[All Fields]))) OR (“musculoskeletal pain”[MeSH Terms] OR (“musculoskeletal”[All Fields] AND “pain”[All Fields]) OR “musculoskeletal pain”[All Fields])) OR (“back pain”[MeSH Terms] OR (“back”[All Fields] AND “pain”[All Fields]) OR “back pain”[All Fields])) OR (lumbosacral[All Fields] AND (“pain”[MeSH Terms] OR “pain”[All Fields]))) OR (discogenic[All Fields] AND (“pain”[MeSH Terms] OR “pain”[All Fields]))) AND (((“epidemiology”[Subheading] OR “epidemiology”[All Fields] OR “prevalence”[All Fields] OR “prevalence”[MeSH Terms]) OR (“epidemiology”[Subheading] OR “epidemiology”[All Fields] OR “incidence”[All Fields] OR “incidence”[MeSH Terms])) OR (“epidemiology”[Subheading] OR “epidemiology”[All Fields] OR “epidemiology”[MeSH Terms]))) AND ((“africa south of the sahara”[MeSH Terms] OR (“africa”[All Fields] AND “south”[All Fields] AND “sahara”[All Fields]) OR “africa south of the sahara”[All Fields] OR (“sub”[All Fields] AND “saharan”[All Fields] AND “africa”[All Fields]) OR “sub saharan africa”[All Fields]) OR (“africa south of the sahara”[MeSH Terms] OR (“africa”[All Fields] AND “south”[All Fields] AND “sahara”[All Fields]) OR “africa south of the sahara”[All Fields]))	253

**Table 3:** Data extraction form

Study details	
Author and publication year	
Study aims and objectives	
Study setting	
Study design	
Sample size ^Male; Female^	
Age group	
Case definition	
Main findings	
Outcomes of interest	

#### Study selection and eligibility

Following deduplication, two independent reviewers will begin in parallel the initial (title and abstract) phase of screening. The titles and abstracts that do not meet the study eligibility criteria will be excluded. Following this initial title and abstract screening phase, a thorough full-text screen of the included articles, at this stage, will be performed, again by two independent reviewers, to assess the eligibility of the article. Studies that do not meet the inclusion criteria will be excluded. A third reviewer will be employed to adjudicate discrepancies between the two reviewers. Following the full text screening, a secondary search of the reference list of all the included studies will be performed for other articles which may not have been identified during database search. The selection of the relevant studies will follow the Preferred Reporting Items for Systematic Review and Meta-Analysis (PRISMA) flow diagram (Fig. 1). Data extraction will be performed on the included articles after the full-text screening by the two independent reviewers.

#### Charting the data

The data from all included studies will be retrieved using a data extraction form (Table 2) by the two independent reviewers. This data extraction form (Table 2) specific to the objectives of the proposed scoping review will be developed by the two reviewers, and this form will be piloted by the two independent reviewers in parallel to test the consistency of the extraction process prior to the commencement of the actual data extraction process. Ten articles will be randomly selected from included studies and will be used for piloting the data extraction form, and if there is need to modify the form based on the results of the pilot study, this will be done prior to its final use. The following information will be extracted from included studies: author and publication year, aims and objectives of the study, study setting, study design, sample size, age group, case definition, and the study main findings. The outcome measures to be analyzed include low back pain prevalence, incidence, mortality, disabilities, comorbidities, risk factors, and the cost estimates. The trends of included studies will also be analyzed to determine if there have been any changes in the distribution of chronic low back pain over time. The data extraction process will be done by two independent reviewers who will discuss any differences until a consensus is reached for the final presentation of data. A coding system will be used to code all the reviewed articles. This is done to keep track of the studies included and excluded during the charting process of the scoping review.

#### Collating, summarizing and reporting the results

The aim of this scoping review is to map the existing evidence on the prevalence, incidence, risk factors, mortality, and cost associated with chronic low back pain among adults in Sub-Saharan Africa and summate the findings of the study articles included. Following completion of the data extraction process, a narrative account of the extracted data from the existing literature will be given using the thematic content analysis [19], where the distribution pattern of chronic low back pain will be noted and analyzed to have a deep appreciation of the study phenomenon. The NVivo 12 data analyses software will be used to extract and categorize data to generate themes from the literature content of included articles [19]. All data that relates to the prevalence of chronic low back pain, incidence of chronic low back pain, risk factors of chronic low back pain, comorbidities and disability related to chronic low back pain, and chronic low back pain estimated cost will be extracted and structured and the emerging themes analyzed with the results critically examined to inspect the relationship between the findings and the study purpose. The meaning of these results will then be considered as they relate to the objectives of the study and the implications thereof for future research, policy-making, and practice will be assessed.

### Quality Appraisal Assessment

All included studies will be assessed for the risk of bias using three reliable quality assessment tools based on the design of the study. The methodological quality of included studies was assessed using a tool adopted from Hoy et al. which has been shown to be a valid tool to assess low back pain epidemiological studies (Table 4). It contained eight items which are sample frame, sample size estimates, randomization used, likelihood of non-response bias, validity of the study instruments, standardization of data collection, use of human body drawings, and if the data was collected directly from the subjects. A score weighting index of 0.2 was attributed to sample size representativeness, non-response bias probability, and randomization. This greater weighting was given to the characteristics that had a higher chance of causing bias in chronic low back pain epidemiological estimates. The remaining five items were rendered an index score weighting of 0.08, which enabled a total score of 1.0. The methodological quality of cost-of-illness studies will assess for the risk of bias using an analytical grid adopted from Costa’s 2012 COI study (Table 5) which contains the following main aspects of COI studies, clear definition of the illness, carefully described epidemiological sources, sufficiently disaggregated costs, description and assessment of activity data source, analytical description of cost values, proper valuation of unit costs, careful description of used methodology, discounted costs, testation of major assumptions, and the consistency of the study results presentation with the methodology of the study [20, 21]. Each item bears the same weight, and the final score will be the sum of all the eleven items. This will be done to make sure that the studies are of moderate to good quality and that the study design is appropriate for the study objectives and to eliminate the risk of bias.

**Table 4:** Methodological quality assessment tool for LBP epidemiological studies

Score weight									
study	0.2	0.08	0.2	0.2	0.08	0.08	0.08	0.08	
	Was the sampling frame a true or close representation of the target population	Was the sample size estimated?	Was some form of random selection used to select sample OR was a census undertaken?	Was the likelihood of non-response bias minimal?	Were data collected directly from the subjects (as opposed to a proxy)?	Had the study instrument that measured the parameter of interest (eg, CLBP prevalence) been tested for reliability and validity?	Was data collection standardised?	Was a human body drawing used	Total score

**Table 5:** Methodological quality assessment of cost-of-illness studies

Study	Was a clear definition of the illness given?	Were epidemiological sources carefully described?	Were the costs sufficiently disaggregated?	Were activity data sources carefully described?	Were activity data appropriately assessed?	Were the sources of cost values analytically described?	Were unit costs appropriately valued?	Were the methods adopted carefully explained?	Were costs discounted?	Were major assumptions tested in a sensitivity analysis?	Was the presentation of study result consistent with the methodology of the study	Total score

## Discussion

The proposed scoping review seek to map existing evidence regarding the distribution (prevalence, incidence and mortality), risk factors, estimated costs associated to chronic low back pain among adults SSA in order to highlight the research gaps in this area. This scoping review will include cohort studies, cross-section studies, and cost of illness studies designs conducted among adults in Sub-Saharan Africa. The studies to be included should have been conducted in English and there will be no date restrictions. Intervention-based studies and randomized controlled clinical trials will be excluded from this review as the data will not be relevant and not be addressing the research question of the review.

This study will be the first study to map the evidence on the distribution of chronic low back pain in Sub-Saharan Africa with estimates on prevalence, incidence, mortality, risk factors, and associated cost. Low back pain is a common public health problem with a significant impact to the society and imposes a financial burden to both the HICs and the LMICs [11]. Low back pain has been referred to as a healthcare enigma because the determinants thereof are unknown in most cases and its normal diagnosis of convenience is non-specific low back pain. According to the Global Burden of Disease 2010, low back pain is the sixth ranking burden among 291 diseases and the cause of more years lived with the disease [10, 22, 23]. Low back pain is the leading cause of disability and activity limitation, resulting in significant production losses at work and demands billions of dollars in medical care expenditure annually [10]. It therefore imposes a significant economic burden on the national heath budgets, further constraining already fragile African health systems.

Mapping out the trends of chronic low back pain over time is essential to predict the future outcomes of the disease. This will help the primary healthcare workers, policy makers, and other stakeholders to take precautionary measures to prevent or minimize the predicted socio-economic burden. The economic forecast of low back pain will help to ensure an efficient healthcare resource allocation, influence cost-benefit, and/or cost-effectiveness analyses studies. Knowing that the mortality trends of low back pain is of paramount importance to healthcare providers. This will serve as a wake-up call for the implementation of programs such as exercise programs for the frail constitution and dietary recommendations, and clinical trials to come up with cost effective interventions.

Epidemiological estimates are essential; knowing which population group is more vulnerable will assist in the design of interventions specific to that population group, for example chronic low back pain is more prevalent among adults of the working class particularly those with a sedentary lifestyle. Health is a human right; therefore, it should be implemented that all employers of sedentary employees are obliged to supply ergonomic chairs for their employees and have gym allowances for exercise programs. All work places should be assessed at regular intervals by safety and health professionals to ensure that all necessary low back pain preventative measures are implemented. By identifying the risk factors of low back pain, this will help primary healthcare providers to implement prevention programs and copying strategies to alleviate the impact of the disease to the society. Mapping out the existing evidence on the prevalence, incidence, mortality, disability, risk factors, and the socio-economic burden of chronic low back pain in SSA will provide evidence-based knowledge gaps, inform future research, and enrich the study findings.

## Conclusion

The proposed scoping review is anticipated to give a reflection on the distribution of chronic low back pain among adults in the SSA region with estimates on prevalence, incidence, mortality with the identification of risk factors, and the associated economic burden. Therefore, the evidence synthesized from this study will help researchers, decision makers, and other stakeholders to inform policy and ensure an efficient allocation of healthcare resources, improving the healthcare system performance and thus improving treatment and reduce mortality associated.

## Supplementary information

**Additional file 1.** PRISMA-ScR Checklist (16).

## Data Availability

All data generated or analyzed during this study will be included in the published systematic review article.

## Abbreviations

SSA: Sub-Saharan Africa
CLBP: Chronic low back pain
KZN: KwaZulu-Natal
HICs: High-income countries
LMICs: Low-middle-income countries
MMQAT: Mixed Methods Quality Appraisal Test
PECOd: Population Exposure Context Outcome design framework
AIDS: Acquired immunodeficiency syndrome
HIV: Human immunodeficiency virus
TB: Tuberculosis
