# Supplementary figures and images for: Mapping evidence on the prevalence, incidence, risk factors and cost associated with chronic low back pain among adults in Sub-Saharan Africa: a systematic scoping review protocol
Source: Syst Rev. 2020 Mar 17;9:57. doi: 10.1186/s13643-020-01321-w (PMC7077148; doi:10.1186/s13643-020-01321-w)

***Appendix A: PRISMA-ScR Checklist (16)***


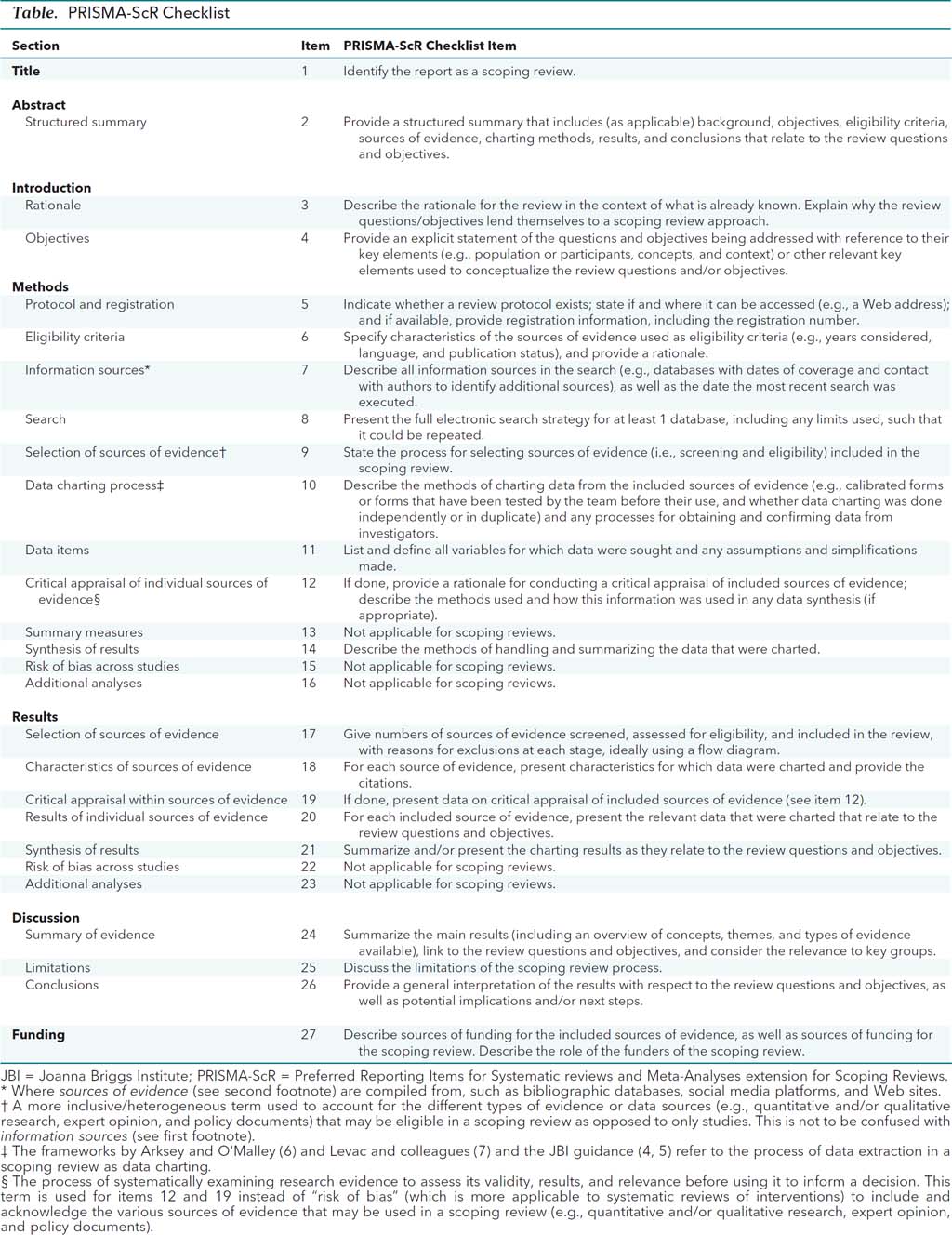

Supplement: Supplementary file 1 — Additional file 1. PRISMA-ScR Checklist (16). [file 13643_2020_1321_MOESM1_ESM.docx]
